# Vascular Smooth Muscle Contractile Function Declines With Age in Skeletal Muscle Feed Arteries

**DOI:** 10.3389/fphys.2018.00856

**Published:** 2018-07-31

**Authors:** John W. Seawright, Harini Sreenivasappa, Holly C. Gibbs, Samuel Padgham, Song Y. Shin, Christine Chaponnier, Alvin T. Yeh, Jerome P. Trzeciakowski, Christopher R. Woodman, Andreea Trache

**Affiliations:** ^1^Department of Health and Kinesiology, Texas A&M University, College Station, TX, United States; ^2^Department of Medical Physiology, Texas A&M University Health Science Center, College Station, TX, United States; ^3^Department of Biomedical Engineering, Texas A&M University, College Station, TX, United States; ^4^Department of Pathology and Immunology, University of Geneva, Geneva, Switzerland; ^5^Department of Veterinary Physiology and Pharmacology, Texas A&M University, College Station, TX, United States

**Keywords:** aging, Rho-kinase, vascular smooth muscle, vasoconstriction, atomic force microscopy

## Abstract

Aging induces a progressive decline in vasoconstrictor responses in central and peripheral arteries. This study investigated the hypothesis that vascular smooth muscle (VSM) contractile function declines with age in soleus muscle feed arteries (SFA). Contractile function of cannulated SFA isolated from young (4 months) and old (24 months) Fischer 344 rats was assessed by measuring constrictor responses of denuded (endothelium removed) SFA to norepinephrine (NE), phenylephrine (PE), and angiotensin II (Ang II). In addition, we investigated the role of RhoA signaling in modulation of VSM contractile function. Structural and functional characteristics of VSM cells were evaluated by fluorescence imaging and atomic force microscopy (AFM). Results indicated that constrictor responses to PE and Ang II were significantly impaired in old SFA, whereas constrictor responses to NE were preserved. In the presence of a Rho-kinase inhibitor (Y27632), constrictor responses to NE, Ang II, and PE were significantly reduced in young and old SFA. In addition, the age-group difference in constrictor responses to Ang II was eliminated. ROCK1 and ROCK2 content was similar in young and old VSM cells, whereas pROCK1 and pROCK2 were significantly elevated in old VSM cells. Aging was associated with a reduction in smooth muscle α-actin stress fibers and recruitment of proteins to cell-matrix adhesions. Old VSM cells presented an increase in integrin adhesion to the matrix and smooth muscle γ-actin fibers that was associated with increased cell stiffness. In conclusion, our results indicate that VSM contractile function declined with age in SFA. The decrement in contractile function was mediated in part by RhoA/ROCK signaling. Upregulation of pROCK in old VSM cells was not able to rescue contractility in old SFA. Collectively, these results indicate that changes at the VSM cell level play a central role in the reduced contractile function of aged SFA.

## Introduction

Aging is associated with a progressive decline in vasoconstrictor responses in central and peripheral arteries (Delp et al., 1995; Kenney and Armstrong, 1996; Dinenno et al., 2002; Qiu et al., 2010). The mechanism responsible for the age-related decrease in vasoconstrictor function has not been fully elucidated but may involve an impaired ability of vascular smooth muscle (VSM) cells to develop contractile tension. This hypothesis is supported by evidence indicating that myogenic constrictor responses in skeletal muscle arterioles declined with age (Muller-Delp et al., 2002; Kang et al., 2009; Ghosh et al., 2015). In addition, agonist-induced vasoconstrictor responses to norepinephrine (NE), phenylephrine (PE), and angiotensin II (Ang II) were impaired in endothelium intact skeletal muscle feed arteries (SFA) from old rats when compared to young rats (Seawright et al., 2016).

Arterial aging results in progressive changes in the mechanical properties of the vessel wall leading to increased wall stiffness and an impaired ability of aged blood vessels to control local blood flow and pressure. At the microscopic level, this translates to decreased responsiveness of VSM and endothelial cells to mechanical stimuli (i.e., mechanosensitivity). This impairment, in turn, induces compensatory hypertrophic or hyperplastic remodeling of aged arteries (Rizzoni et al., 2000; Najjar et al., 2005; Briones et al., 2007). The discrete VSM cell mechanical properties and their ability to adapt to external mechanical signals (e.g., blood pressure and flow) directly contribute to maintaining vessel tone (Martinez-Lemus et al., 2005b; Martinez-Lemus et al., 2009). An impaired ability to properly regulate vascular tone through appropriate vasoconstrictor responsiveness may lead to orthostatic intolerance, impaired blood flow distribution, and reduced exercise capacity in the elderly (Davy et al., 1998; Musch et al., 2004).

Vascular smooth muscle cells play an integral role in regulating matrix deposition and vessel wall contractility via interaction between the actomyosin contractile unit and adhesion structures formed at the cell membrane that mechanically link the cell to the matrix (Lakatta, 2003; Saphirstein et al., 2013). The actin cytoskeleton is responsible for maintaining cell shape and provides the platform for the distribution of mechanical signals throughout the cell (Olson and Nordheim, 2010). Dynamic rearrangement of actin stress fibers is necessary to redistribute physical forces needed for cell contraction that enables cell adaptation to the extracellular microenvironment (Chen et al., 2013). Thus, external mechanical stresses are balanced by intracellular counter-forces provided by the actomyosin apparatus that forms stress fibers (Wang et al., 2001; Ingber, 2006). This mechanical load-bearing cell-matrix interaction is key to maintaining the contractile state of resistance arteries. Most studies to date on arterial aging have focused on the role played by endothelial dysfunction or changes in the extracellular matrix, and less on the contribution of VSM cells that control vessel tone (Herrera et al., 2010; Duca et al., 2016). However, there is emerging interest in the role VSM cells play in regulating vessel wall stiffness (Kohn et al., 2015; Sehgel et al., 2015).

Aging also induces an increase in the activation of RhoA, a Rho family GTPase protein involved in cell contraction, in aorta of old animals (Miao et al., 2001). Our group (Lim et al., 2012; Sreenivasappa et al., 2014), and others (Huveneers et al., 2015) have shown that the RhoA pathway induces increased cellular contractility and adhesion by activating downstream effectors, including Rho-kinase (ROCK). In turn, ROCK regulates myosin activity by promoting myosin light chain phosphorylation and increasing actomyosin contractile function.

In the present study we tested the hypothesis that VSM contractile function in SFA declines with age. In addition, we investigated the role of the RhoA signaling pathway in modulation of VSM contractile function in SFA.

## Materials and Methods

### Animals

Before initiating this study, approval was received from the Texas A&M University Institutional Animal Care and Use Committee. Young (4 months) and Old (24 months), male Fischer 344 rats were obtained from the National Institute on Aging (NIA) and housed at the Texas A&M Comparative Medicine Program Facility. All rats were housed under a 12:12 h light-dark cycle and provided food and water *ad libitum*. The rats were examined daily by Animal Care Facility veterinarians and by study investigators.

### Isolation and Cannulation of Soleus Muscle Feed Arteries

#### Isolation of Soleus Muscle Feed Arteries

Soleus muscle feed arteries were isolated as previously described (Woodman et al., 2007; Trott et al., 2009; Seawright et al., 2014). Briefly, rats were anesthetized using an intraperitoneal injection of Ketamine (80 mg/kg body weight) and Xylazine (5 mg/kg body weight) and plane of anesthesia was verified by an unresponsive toe-pinch. The soleus-gastrocnemius-plantaris muscle complex was then removed from each hindlimb and placed in cold (4°C) MOPS-buffered physiological saline solution (PSS) containing: 145 mM NaCl, 4.7 KCL, 2 mM CaCl_2_, 1.17 mM MgSO_4_, 1.2 mM NaH_2_PO_4_, 5 mM glucose, 2 mM pyruvate, 0.02 mM EDTA, and 25 mM MOPS (pH 7.4). SFA were isolated, dissected free, and placed in a Lucite chamber containing MOPS-PSS (pH 7.4 at 4°C) for cannulation. Rats were euthanized by excising the heart.

#### Cannulation of Soleus Muscle Feed Arteries

To isolate the role of VSM in constrictor responses we used SFA with endothelium removed (i.e., denuded SFA). Specifically, one end of each SFA was cannulated with a glass micropipette and secured with surgical thread. The endothelial lining was removed from each SFA by passing 5 ml of air through the lumen of the vessel and flushing the artery with PSS-albumin. The other end of the SFA was then cannulated with a second glass micropipette and secured with surgical thread. After both ends of the SFA were cannulated, each glass micropipette was connected to a pressure reservoir containing MOPS-PSS with albumin (1 g/100 ml). The height of each reservoir was adjusted to set intraluminal pressure in each feed artery to 60 cm H_2_O (1 mm Hg = 1.36 cm H_2_O). SFA were checked for leaks by verifying that intraluminal diameter was maintained after closing the pressure reservoirs. SFA determined to be leak free were subsequently pressurized at 90 cm H_2_O for 1 h at 37°C. An intraluminal pressure of 90 cm H_2_O was selected based on previous studies (Woodman et al., 2007; Seawright et al., 2014) and is representative of the pressure thought to be present in a rat SFA at rest (Williams and Segal, 1993). Endothelial cell denudation was confirmed by the lack of vasodilation (<5%) following addition of acetylcholine (ACh, 3 × 10^-4^ M).

### Assessment of Soleus Muscle Constrictor Responses

Vasoconstrictor responses of denuded arteries were assessed by measuring the change in diameter of SFA in response to cumulative, increasing, whole log concentrations of NE (10^-9^ × 10^-4^ M), PE (10^-9^ × 10^-4^ M), and angiotensin II (Ang II: 10^-11^ × 10^-7^ M). NE and PE were purchased from Sigma-Aldrich (St. Louis, MO, United States) and Ang II was acquired from Bachem (Torrance, CA, United States).

To evaluate the contribution of ROCK to VSM contractile function, smooth muscle constrictor responses were assessed in the absence or presence of a ROCK selective inhibitor (Y27632, Bachem, Torrance, CA, United States). Y27632 (10^-6^ M) was added to the vessel bath 20 min prior to assessing smooth muscle constrictor responses (Seawright et al., 2016). Y27632 was purchased from Sigma-Aldrich (St. Louis, MO, United States). Two SFA were studied from each young and old rat. In one SFA, NE, PE, and Ang II-induced constrictor responses were assessed in the absence of the ROCK inhibitor, while in the second SFA, same constrictor responses were assessed in the presence of the ROCK inhibitor.

All values are presented as mean ± SE. One-way ANOVA was used to assess differences in body weight and maximal passive diameter between young and old groups. Two-way ANOVA with repeated measures on one factor (concentration) was used to determine differences in constrictor responses to NE, PE, or Ang II between young and old groups. Concentration-response data were expressed as percent constriction and were calculated as [(Db–Dc)/Db] × 100, where Dc is the measured diameter for a given concentration, and Db is the baseline diameter measured before each concentration response curve (Park et al., 2012). When a significant *p*-value was obtained, *post hoc* analyses were performed using Duncan’s Multiple Range Test. Statistical significance was evaluated at *p* < 0.05.

### Vascular Smooth Muscle Cell Isolation and Cell Culture

Vascular smooth muscle cells were explanted from SFA isolated from young and old Fischer 344 rats. SFA were handled in aseptic conditions and thoroughly washed in sterile saline solution. SFA were cleaned of fatty tissue and mounted in a 35 mm cell culture dish containing VSM cell medium (Dulbecco’s Modified Eagle Medium supplemented with 10% fetal bovine serum and 10 mM HEPES (Sigma-Aldrich, St. Louis, MO, United States), 2 mM L-glutamine, 1 mM sodium pyruvate, 100 U/ml penicillin, 100 μg/ml streptomycin, and 0.25 μg/ml amphotericin B) and set in an incubator at 37°C with 5% CO_2_. After 7 days the tissue was removed and smooth muscle cells attached to the dish were left to grow until they reached 90% confluence, at which point they were passed to maintain the cell culture. Low passage cells cultured in the same VSM cell medium were used for experiments. All reagents were purchased from Invitrogen (Carlsbad, CA, United States), unless otherwise specified.

### Assessment of ROCK Activity

ELISA assays were used to quantify total and activated ROCK protein for both isoforms, ROCK 1 and ROCK 2. Samples prepared from protein cell lysates at a concentration of 500 μg/ml were loaded in each well, and experiments were run in duplicate following the manufacturer’s protocol. Test kits from MyBioSource (San Diego, CA, United States) were used for total ROCK 1 and 2, and pROCK 1 and 2. The optical density for standards and samples was measured at 450 nm using a Victor X3 2030 plate reader (PerkinElmer, Waltham, MA, United States) and standard curve fitting was performed. Data were normalized to compensate for differences in the concentration of protein in the sample. Old and young samples within the same assay were tested for statistical significance with ANOVA. Statistical computations were performed using R software v. 3.3.2 (The R Foundation for Statistical Computing). Significance was evaluated at *p* < 0.05.

### Tri-Dimensional Contractility Assay

#### Collagen Gel Preparation

Collagen type 1 (Rat tail collagen, Corning, Corning, NY, United States) was prepared using the manufacturer’s protocol. Cells in suspension were mixed with the collagen get at a final concentration of 150,000 cells/ml and 3.5 mg/ml collagen. The collagen-cell gel was further adjusted to pH 7.3–7.6, and 250 μl was added to each well of a 48-well plate. The plate was incubated at room temperature for 45 min, after which 500 μl of VSM cell medium was added to each well and incubated at 37°C with 5% CO_2_ for 24 h. The collagen-cell gel was further fixed in 4% paraformaldehyde and 5% sucrose in Dulbecco’s Phosphate Buffered Saline (DPBS) (Morris et al., 2017).

#### Imaging of Collagen Gels by Non-linear Optical Microscopy

Vascular smooth muscle cells suspended in type I collagen as described above were imaged with a custom-built non-linear optical microscope as previously described (Larson and Yeh, 2006). Briefly, 10 femtosecond (fs) ultrashort pulses at 800 nm from a Ti:Sapphire oscillator (Femtolasers, Vienna, Austria) were pre-compensated with double-chirped mirrors (Femtolasers, Vienna, Austria), coupled into an upright microscope (Carl Zeiss, Thornwood, New York, United States) by x-y scanners (Cambridge Technologies, Cambridge, MA, United States), and focused with a 1.0 NA, 20× objective (Carl Zeiss, Thornwood, NY, United States). Second harmonic generation (SHG) and two-photon excited fluorescence from samples were collected by the same objective and separated with a dichroic mirror (430 nm long pass) and bandpass filters (405/20 nm for collagen I SHG and 450/60 nm for cellular auto-fluorescence) before being focused onto photomultiplier tube detectors (Hamamatsu, Bridgewater, NJ, United States). Voxel dimensions were 0.5 μm × 0.5 μm × 0.5 μm.

#### Fluorescence Quantification

Maximum intensity projection images were generated from 3D image stacks for qualitative reference with FIJI and Vaa3D (Peng et al., 2010). 2D xy images of raw photon counts from individual optical sections were used for quantitative analysis with a custom program in Matlab (Mathworks, Natick, MA, United States). The distribution of collagen fiber angle orientation was determined using a Fast Fourier Transform algorithm that has been previously described (Chaudhuri et al., 1987) and applied to the SHG images. This fiber angle distribution was characterized by the alignment index (AI), defined as the ratio of the fiber distribution within 20° of the dominant fiber angle to the value found within the same range of a random distribution (Bai et al., 2014). Theoretically, an AI of 1.0 would indicate a random fiber distribution, while completely parallel fibers would give an AI of 4.55. The experimentally measured range does not typically span the entire theoretical range, and the AI values measured here are similar to those reported previously in other culture systems by us and other groups (Bai et al., 2014; Mohammadi et al., 2014).

### Assessment of VSM Cell Architecture

#### Immunofluorescence Staining

Vascular smooth muscle cells cultured as described above in glass bottom dishes (MatTek, Ashland, MA, United States) were fixed after 24 h by immersion in 2% paraformaldehyde in DPBS followed by washing in a glycine buffer. Cells were incubated overnight at 4°C with primary antibody smooth muscle α-actin (SMα-actin), pFAK-397 or vinculin (Sigma, St. Louis, MO, United States) diluted in a sodium citrate buffer containing BSA and Triton X (Sun et al., 2005). Vinculin was used as a general marker for focal adhesions, while pFAK397 was chosen because it is known to have an important role in adhesion activation and cell migration. SMα-actin was used as a marker for stress fiber formation. After washing, cells were incubated with Alexa 488 or 568 secondary antibody (Invitrogen, Carlsbad, CA, United States) for 1 h at room temperature, followed by another round of washing, and then immediate imaging in DPBS. Phalloidin staining (1 h incubation at room temperature) was also performed to evaluate all filamentous actin in the cell.

For smooth muscle γ-actin (SMγ-actin) staining, cells were first fixed with 1% paraformaldehyde in DPBS followed by permeabilization with cold methanol (Arnoldi et al., 2013; Chaponnier and Gabbiani, 2016). Cells were then incubated overnight at 4°C with a SMγ-actin primary antibody in DPBS. After washing, cells were incubated with Alexa 488 secondary antibody for 1 h at room temperature, followed by another round of washing, and then immediate imaging in DPBS.

#### VSM Cell Imaging

Cell imaging experiments were performed on the integrated microscope system as previously described (Trache and Lim, 2009). A PLAN APO 60× oil 1.45 NA TIRF objective lens was used for both total internal reflection fluorescence (TIRF) and spinning-disk confocal microscopy. 3D confocal images were acquired as stacks of 20 planes at a 0.25 μm step size with an exposure time of 100 ms, and are presented as xy projections. TIRF images were also acquired with an exposure time of 100 ms.

#### Fluorescence Quantification

Evaluation of protein area in fluorescence images was performed by using the masking tool and image statistics tools in the SlideBook software (Intelligent Imaging Innovations, Denver, CO, United States) as previously described (Lim et al., 2010). Briefly, protein area for vinculin and pFAK at focal adhesions was determined from TIRF images. Projections of confocal images were used to measure actin area throughout the cell. To statistically compare a large number of cells, fluorescence protein area was normalized to total cell area for each cell before statistical analysis. ANOVA was used for statistical analysis and differences were considered significant at *p* < 0.05.

### Adhesion Force Spectroscopy

Atomic force microscopy (AFM) experiments were performed using an integrated microscope system as previously described (Trache and Lim, 2009). Briefly, the AFM experiments were performed with unsharpened silicon nitride cantilevers (MLCT, Bruker Nano Surfaces Inc., Santa Barbara, CA, United States) functionalized with fibronectin (FN, Invitrogen, Carlsbad, CA, United States). The AFM probe was mounted on the glass holder, and washed well with DPBS. Then, the probe was incubated for 5 min with polyethylene glycol at 10 mg/ml to cross-link FN onto the AFM probe. The probe was washed again and subsequently incubated for 1 min with 1 mg/ml FN (Trache and Meininger, 2008).

Force curves were acquired by driving the cantilever to touch and retract from the cell surface over a known predefined distance in the *z*-axis. All force curves were acquired at positions midway between the nucleus and the edge of the cell. Experiments were performed in duplicates, for a total of ∼4,000 individual force curves. The adhesion force was calculated by multiplying the change in deflection height associated with the unbinding event by the spring constant of the cantilever (12.2 ± 0.4 pN/nm). The local cell stiffness at the point of contact was calculated as Young’s modulus of elasticity, by fitting the approach curve between the initial point of cell contact and point of maximum probe displacement with Sneddon’s modified Hertz model (Trache et al., 2005). Distributions of force or elasticity measurements were generated using normal reference bandwidths and Gaussian kernel functions (Silverman, 1986) in NForcer software (Trzeciakowski and Meininger, 2004). PeakFit software (version 4.11, Systat Software Inc.) was used to accurately estimate the peak value and associated confidence intervals for each distribution. Peaks whose confidence intervals did not overlap were considered significantly different (*p* < 0.05) (Venables and Ripley, 1994).

## Results

### Characteristics of Rats and SFA

Young rats (345 ± 5 g) weighed significantly less than old rats (423 ± 15 g). Maximal passive diameters of SFA were not different between young (140 ± 11 μm) and old (138 ± 15 μm) SFA.

### Smooth Muscle Constrictor Responses Are Reduced in Aged SFA

**FIGURE 1:**
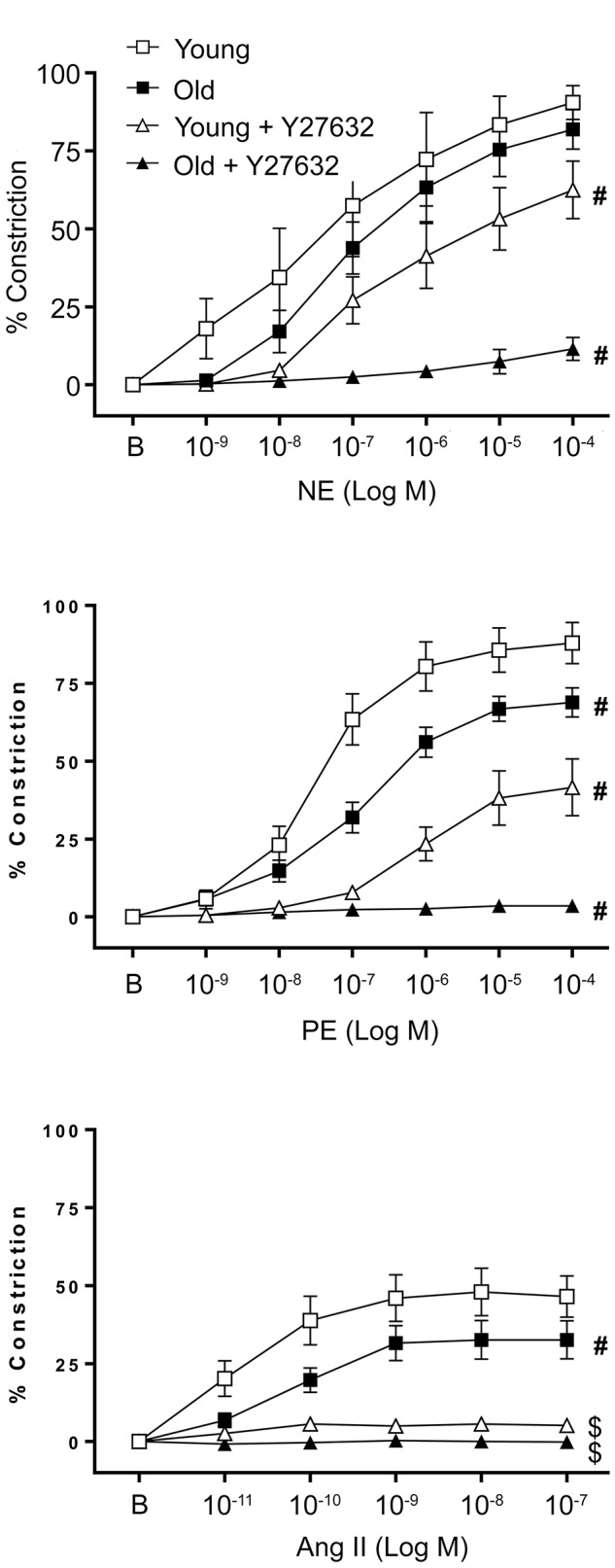
Effect of aging on vascular smooth muscle contractile responses to NE, PE, and Ang II was assessed in denuded SFA. To evaluate the role of ROCK, VSM constrictor responses were further assessed in the presence of Y27632. *N* = 6–12 rats per group. B represents the baseline diameter at a pressure of 90 cm H_2_O before the first dose of NE, PE, or Ang II. Data shown are mean ± SE. Significance was evaluated at *p* < 0.05. ^#^Concentration-response curve was significantly different from all other curves; ^$^Concentration-response curve was significantly different from young and old SFA in the absence of Y27632.

To determine the effect of aging on vasomotor function of VSM cells in a tissue model, SFA isolated from young and old animals were cannulated, denuded to remove endothelium, and exposed to three different contractile agonists (NE, PE, and Ang II). **Figure 1** shows that all three agonists induced a concentration-dependent constriction of denuded SFA from young and old rats. Smooth muscle contractile responses to NE were not different between young and old SFA, whereas constrictor responses to PE were significantly impaired in old SFA relative to young SFA. These results suggest that aging selectively impaired α1 adrenergic receptor-mediated contractile responses in VSM. Furthermore, stimulation of the AT1 receptors by Ang II induced constriction in both young and old SFA. The Ang II contractile response was also impaired in aged SFA compared to young SFA. Taken together, these results suggest that VSM contractility declines with age in SFA and the responses may be receptor specific.

### Rho-Kinase Activation Is Unable to Rescue the Impaired Functional Constrictor Responses in Aging

To evaluate the contribution of ROCK activation to functional smooth muscle contraction in a tissue model, constrictor responses of denuded SFA were assessed in the absence or presence of a ROCK inhibitor (Y27632). In the presence of Y27632, smooth muscle constrictor responses to NE, PE, and Ang II were significantly reduced in both young and old SFA (**Figure 1**). While in old denuded arteries the constrictor responses were almost abolished, young arteries presented a differential response to constrictors in the presence of Y27632. Thus in NE treated vessels Y27632 induced a moderate reduction, while in PE treated vessels it produced a robust reduction in VSM constrictor response. In addition, since Y27632 inhibition eliminated constrictor responses to Ang II in both young and old groups, a subset of SFA was washed with warm PSS to remove Y27632 and the Ang II concentration response curve was repeated. Following removal of Y27632, constrictor responses to Ang II were restored, providing evidence that SFA were viable following exposure to Y27632 (results not shown).

**FIGURE 2:**
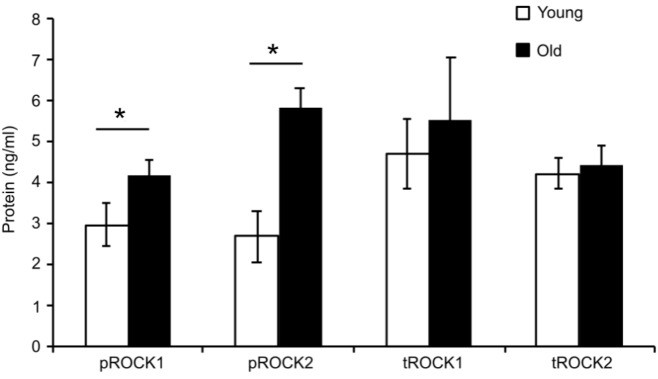
Elisa assay values for total and phospho-ROCK proteins. While total ROCK does not change with age, there was a significant increase in old cells for both isoforms in the active form pROCK1 and pROCK2. *N* = 4. Data shown as mean ± SD. ^∗^Significance was evaluated at *p* < 0.05.

To determine whether ROCK activity was altered in aged SFA, quantitative ELISA assays were used to determine the level of total ROCK as well as activated ROCK for both isoforms (ROCK1 and ROCK2) in cells isolated from both young and old rats. **Figure 2** shows that there was no difference between total ROCK 1 and 2 in young and old cells; however, there was a significant increase in both active forms pROCK1 and 2 in old cells. These results show that upregulation of Rho-kinase in its active form is not able to rescue the reduced contractility state of the old cells.

### Aging Alters Contractile Properties of VSM Cells

**FIGURE 3:**
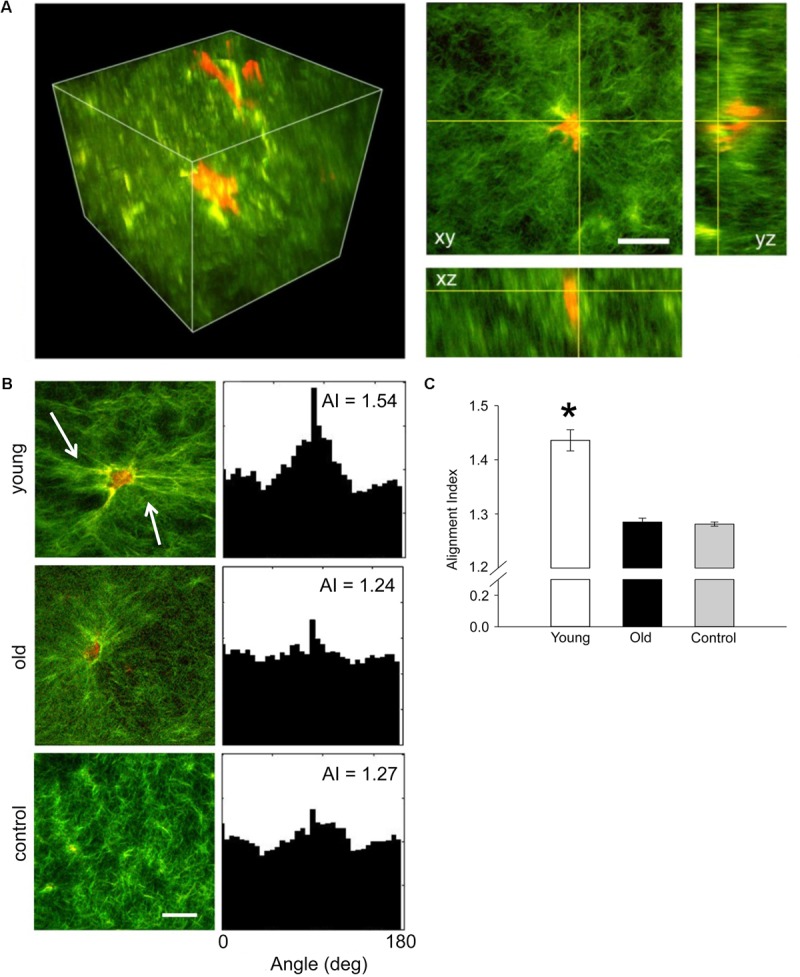
**(A)** Representative three-dimensional volumetric imaging of the collagen gel and 2D projections in xy, xz, and yz planes. Collagen gel was pseudo-colored in green (SHG signal), while auto-fluorescence signal from VSM cells was pseudo-colored in red. **(B)** Representative projections of the 3D volumetric images and their alignment angle distributions and alignment index (AI), respectively. Young VSM cells exert force on the collagen matrix inducing higher remodeling of the collagen fibers (see arrows). In contrast, old VSM cells have reduced local matrix remodeling. **(C)** The distribution of collagen fiber angle alignment index for young cells shows a significant increase in respect to control (collagen gel without cells), while no change was recorded for old cells. *N* = 8–10 tri-dimensional volumetric images per condition. Scale bar represents 50 μm. Data shown as mean ± SD. ^∗^Significance was evaluated at *p* < 0.05.

Contractile function of VSM cells isolated from young and old SFA was tested by embedding the cells in collagen I hydrogels. Three-dimensional imaging by SHG non-linear microscopy (Bai et al., 2014) allowed acquisition of image stacks at steps of 0.5 μm to reconstruct volumetric rendering as shown in **Figure 3A**. Thus, cell-matrix interactions were visualized in three-dimensions. **Figure 3B** shows that VSM cells (red) from young rats exert force on the collagen matrix (green) inducing extensive collagen fiber remodeling (**Figure 3**, see arrows), while no significant fiber remodeling was observed in collagen gels containing cells from old rats. Quantitative assessment of collagen matrix remodeling was performed by defining an AI parameter of matrix fibers within the image frame. The AI of matrix fibers increased for hydrogels containing young cells, while no change was measured for hydrogels containing old cells with respect to control. These results suggest that aged cells are not able to generate the force needed to induce matrix remodeling and maintain arterial contractility.

### Morphology of VSM Cells Isolated From SFA Is Age-Dependent

**FIGURE 4:**
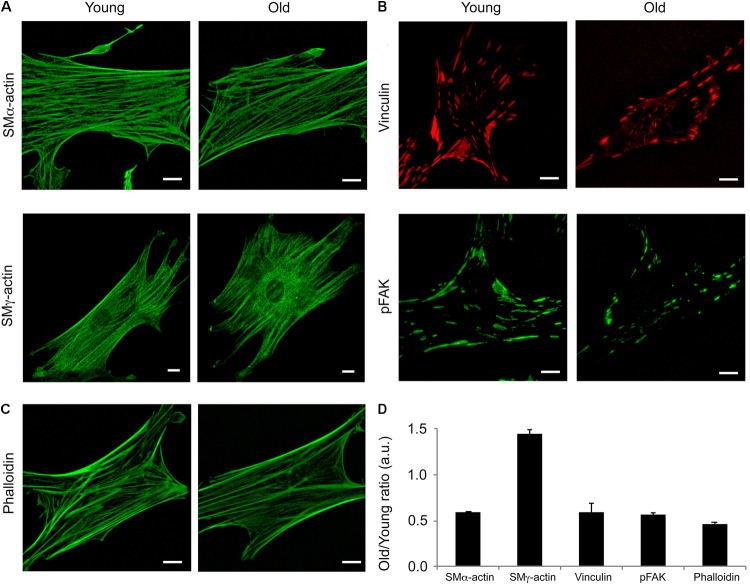
Age effects on the architecture of VSM cells. Representative **(A)** confocal images of SMα- and SMγ-actin, **(B)** TIRF images of vinculin and pFAK-Y397, and **(C)** confocal images of phalloidin in VSM cells isolated from young and old Fischer 344 rats. SMα-actin forms actin bundles at cell edges, while SMγ-actin forms finer fibers toward the cell interior without strong fibers at cell edges. Scale bar represents 10 μm. **(D)** Quantitative analysis of fluorescence images are presented as old to young ratios of relative protein area to respective cell area. *N* = 7–11 cells per condition. Values different from a ratio of 1 are statistically significant (*p* < 0.05).

Isoform specific actins have different structural distributions and roles in the VSM cells (Kim et al., 2008). Moreover, it has been shown that aging affects cytoskeletal and adhesion proteins expression in other vascular beds (Qiu et al., 2010). To determine the contribution of two major actin isoforms known to be present in smooth muscle, we performed confocal imaging of young and old fixed VSM cells isolated from SFA and stained with specific SMα-actin and SMγ-actin antibodies. As shown in **Figure 4A**, young cells had a greater density of SMα-actin stress fibers (i.e., contractile phenotype), while the old cells presented less stress fibers (i.e., senescent phenotype). In contrast, a well-organized arrangement of SMγ-actin fibers was present in cells isolated from old animals, but not in cells isolated from young animals. In addition, SMα-actin forms actin bundles at cell edges, while SMγ-actin forms finer fibers toward the cell interior without strong fibers at cell edges. Quantitative analysis showed that the assembly of α-actin stress fibers decreased twofold, while SMγ-actin fibers increased significantly in old cells. However, isoform-independent staining using phalloidin showed that there was a 40% decrease in total actin fibers in cells from old animals in comparison with young animals (**Figures 4C,D**). These results suggest that aging induces major reorganization of actin cytoskeleton characterized by a reduction in total filamentous actin in cells isolated from SFA.

To further investigate if aging affects VSM cell adhesion to the matrix, immunofluorescence staining for vinculin and pFAK397 followed by TIRF imaging (**Figure 4B**) was performed on young and old cells. TIRF imaging showed that young cells presented well-defined streak-like focal adhesions rich in vinculin (red) and pFAK397 (green) distributed all over the basal cell area, while the old cells presented smaller focal adhesions distributed mostly toward the cell edges. Quantitative analysis of fluorescence images (**Figure 4D**) showed that the protein area for both focal adhesion proteins (pFAK and vinculin) was two-fold lower in old cells compared with young cells. Taken together, these data show that aging is associated with a reduction in proteins essential for VSM contraction and focal adhesion formation.

### Aging Alters Functional Properties of VSM Cells

**FIGURE 5:**
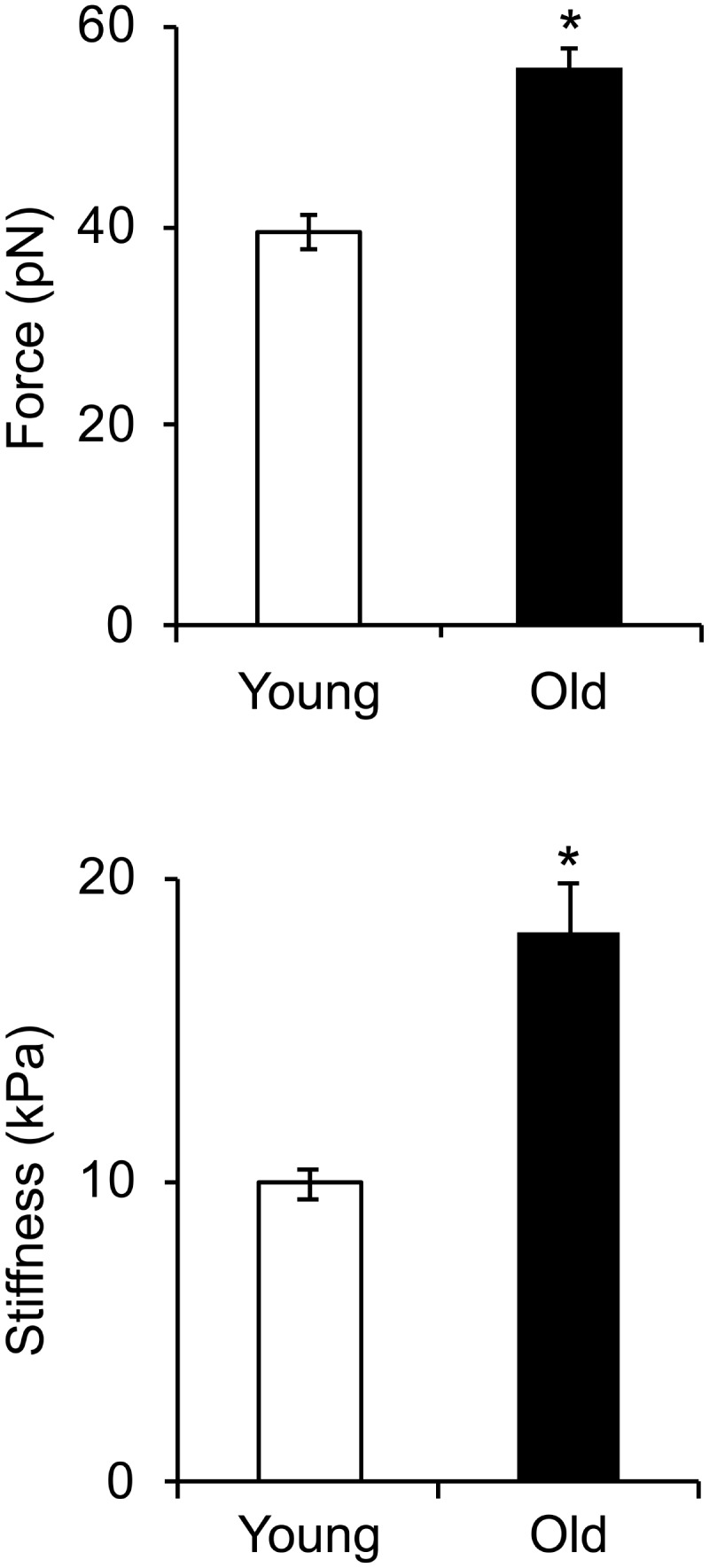
Integrin α_5_β_1_-fibronectin adhesion force spectroscopy measurements. Cell stiffness and adhesion force measurements were performed on young and old cells. Cell stiffness and adhesion force of integrin α_5_β_1_ binding to fibronectin significantly increased with age. *N* ∼ 4,000 individual measurements for each condition. ^∗^Significance was evaluated at *p* < 0.05.

Given the reduced expression of cytoskeletal and focal adhesion proteins with age, we asked if these changes affect integrin mediated adhesion to the matrix and cell stiffness. To address this question, we first determined the mechanosensitive response of integrin α_5_β_1_ adhesion to fibronectin from single ligand-receptor adhesion force spectroscopy measurements using the AFM. Our results showed a significant increase in α_5_β_1_-FN adhesion force in VSM cells isolated from old animals by comparison with young (**Figure 5**). Moreover, a 20% increase in adhesion probability of α_5_β_1_ integrin binding to FN in old cells suggests that aging activates endogenous α_5_β_1_ integrin expression. In addition, our results showed that cell stiffness measured in old cells is significantly higher than in young cells. Taken together, these results suggest that aging increases integrin α_5_β_1_ functional adhesion to the matrix and cortical cell stiffness.

## Discussion

Aging is associated with a progressive decline in vasoconstrictor responses in central and peripheral arteries in humans and animals (Delp et al., 1995; Kenney and Armstrong, 1996; Dinenno et al., 2002; Qiu et al., 2010). Arterial stiffening, a hallmark of the aging process, is a mechanical property of the vessel wall that occurs throughout the vascular tree. Physiologically, increased arterial stiffness contributes to increased blood pressure. Our previous studies demonstrated that vasoconstrictor responsiveness declines with age in intact SFA (Seawright et al., 2016), an artery that plays an important role in regulating blood flow to the soleus muscle (Williams and Segal, 1993). However, since all studies were completed on SFA with intact endothelium, the role of VSM in the age-related decrease of constrictor function was not independently assessed. Therefore, in the present study we directly evaluated the role of VSM in the age-related decrease of the constrictor function. To isolate the role of VSM, experiments were performed in denuded (endothelium removed) SFA isolated from young and old Fischer 344 rats, as well as VSM cell cultures. The present study shows that changes at the VSM cell level play a central role in the reduced contractile function of aged SFA. Our results showed that: (a) age-induced decrease in SFA contractile function was mediated in part by RhoA/ROCK signaling; (b) upregulation of ROCK in old VSM cells was not able to rescue SFA contractility; and (c) aged VSM cells were not able to generate the force needed to induce matrix remodeling due to a reduction in SMα-actin essential for VSM cell contraction, increased stiffness, and altered formation of cell-matrix adhesions.

Our results from functional experiments performed on denuded arteries showed that constrictor responses to PE and Ang II were significantly impaired in denuded old SFA relative to denuded young SFA indicating that VSM contractility was impaired in the aged SFA (**Figure 1**). However, NE-induced constrictor responses were preserved in denuded old SFA, which is in contrast to previous studies using endothelium-intact arteries showing that NE-induced constriction declined with age in SFA (Seawright et al., 2016) and gastrocnemius 1A arterioles (Gittemeier et al., 2017). However, these findings are in good agreement with data from a different study which also showed preservation of NE-induced vasoconstriction in denuded soleus and gastrocnemius 1A arterioles (Donato et al., 2007). We propose that the decrease in NE-induced constriction reported previously in aged, intact SFA are endothelium-dependent whereas the age-induced decline in constrictor responses to PE and Ang II are the result of impaired VSM contractile function.

In addition, our studies in denuded SFA revealed that VSM constrictor responses to the α-1 adrenergic selective agonist PE were impaired, whereas constrictor responses to NE which activates α-1 and α-2 adrenergic receptors were preserved. Taken together, these data suggest that α-2-mediated constriction did not change with aging. This interpretation is consistent with previous studies revealing that constrictor responses to PE declined with age in feed arteries perfusing the gluteus maximus muscle whereas constrictor responses to an α-2 agonist (UK 14304) were preserved (Sinkler and Segal, 2014). Alternatively, preserved VSM contractile responses to NE in old SFA may have been due to enhanced NE stimulation of dilatory beta adrenergic receptors on VSM. However, since previous studies by Donato et al. (2007) using soleus muscle first order arterioles revealed that beta adrenergic-mediated vasodilation was reduced with aging in denuded soleus 1A arterioles, this explanation seems unlikely. Future studies will be needed to investigate the mechanism by which α-2 adrenergic receptors contribute to the preservation of aged adrenergic contractile responses. In either case, our results suggest that aging selectively impaired α-1 adrenergic receptor-mediated smooth muscle contraction in SFA.

Rho-kinase, a downstream effector of RhoA, plays a primary role in regulating cellular contractility. Our previous data showed that RhoA-induced contractility engages ROCK and further regulates VSM cell mechanosensitivity in the microvasculature (Lim et al., 2012; Sreenivasappa et al., 2014). To assess the role of ROCK signaling on aged VSM contractile function, constrictor responses were assessed in denuded SFA in the absence and presence of Y27632 (**Figure 1**). Our results indicated that VSM constrictor responses to NE, PE, and Ang II were reduced in both young and old SFA in the presence of Y27632, indicating that ROCK signaling contributed to VSM contractile responses. In addition, constrictor responses to Ang II were abolished by Y27632 in both young and old arteries suggesting that Ang II-induced constrictor responses were mediated primarily via signaling through ROCK. Collectively, these results indicate that VSM contractile function in aging is, in part, regulated by RhoA/ROCK signaling pathway. This finding was further supported by ELISA assay (**Figure 2**) that showed an increase in pROCK in aged cells, but no difference in total ROCK protein.

The mechanism by which aging impairs Ang II-induced VSM cell contractility is not clear; however, Yoon et al. (2016) reported previously that circulating levels of Ang II were higher in old mice when compared with young mice. Therefore, it is possible that an age-induced increase in Ang II in the old rats used in the present study could have resulted in AT1 receptor desensitization, which in turn increased ROCK phosphorylation in an attempt to preserve VSM cell contractile function.

The contractile properties of VSM cells are determined by their cytoskeleton network. Changes in actin cytoskeleton directly affect important cellular functions, including cell contraction and adhesion. In young arteries, VSM cells within the vessel wall present a contractile phenotype which is specific to their elongated, spindle shape differentiated state. However, in aging, VSM cells dedifferentiate toward a synthetic phenotype characterized by impaired contractility, due in part to age-induced changes in essential contractile and adhesion proteins. Isoform specific actins have different structural distributions and roles in VSM cells (Kim et al., 2008; Dugina et al., 2009). For example, SMα-actin cytoskeleton is directly involved in stress fiber formation of the actomyosin apparatus, while SMγ-actin does not directly participate in cell contraction and is rather present toward the central part of the cell (Arnoldi et al., 2013). Our studies showed a significant decrease in SMα-actin fibers, but an increase in SMγ-actin fibers in old cells (**Figures 4A,D**) associated with increased cortical stiffness (**Figure 5**). These findings are in good agreement with Li et al. (1997) who showed that aging is associated with a decrease in SMα-actin in aged aortas from Fischer 344 rats. We propose that the reduction in SMα-actin fibers in old VSM cells lead to a reduced contractile capacity of these cells, while the increase in SMγ-actin fibers may contribute to increased cell stiffness.

Extracellular matrix stiffening induces alteration of cell-matrix adhesions and further activation of intracellular pathway leading to actomyosin contraction. In large conduit vessels (i.e., aorta) it has been shown that aging is associated with an increase in VSM cell stiffness that corresponds to an increase in β_1_ integrin (Qiu et al., 2010), as well as an increase in collagen deposition and a decrease in elastin fibers in the aortic wall (Zhang et al., 2016). Our results also showed an increase in α_5_β_1_ integrin adhesion to the matrix (**Figure 5**), but a decrease in FAK activation at cell-matrix adhesions (**Figures 4B,D**). FAK phosphorylation is associated with enhanced migration (Sieg et al., 1999; Hauck et al., 2002), while integrin α_5_β_1_ is mostly associated with focal adhesion maturation (Danen et al., 2002). These results were further supported by the inability of old VSM cells embedded in a 3D collagen matrix to interact with the extracellular matrix and exert forces able to remodel the collagen fibers (**Figure 3**).

We suggest that the reduced presence of the main players of the contractile apparatus (i.e., SMa-actin) and alterations in cell-matrix adhesions are responsible for the reduced contractility. In support of our hypothesis, our previous publication (Seawright et al., 2016) has shown that lysophosphatidic acid (LPA), a RhoA activator that subsequently activates ROCK, partially restores SFA contractility *in vitro*. However, LPA is known to induce stress fiber formation and increases recruitment of proteins at cell-matrix adhesions (Lim et al., 2010). Thus, in aging, the decrease in SMα-actin stress fiber formation and protein recruitment at cell-matrix adhesions induces compensatory effects by up-regulating ROCK and SMγ-actin which increases cell stiffness without regaining contractile properties of the cell.

**FIGURE 6:**
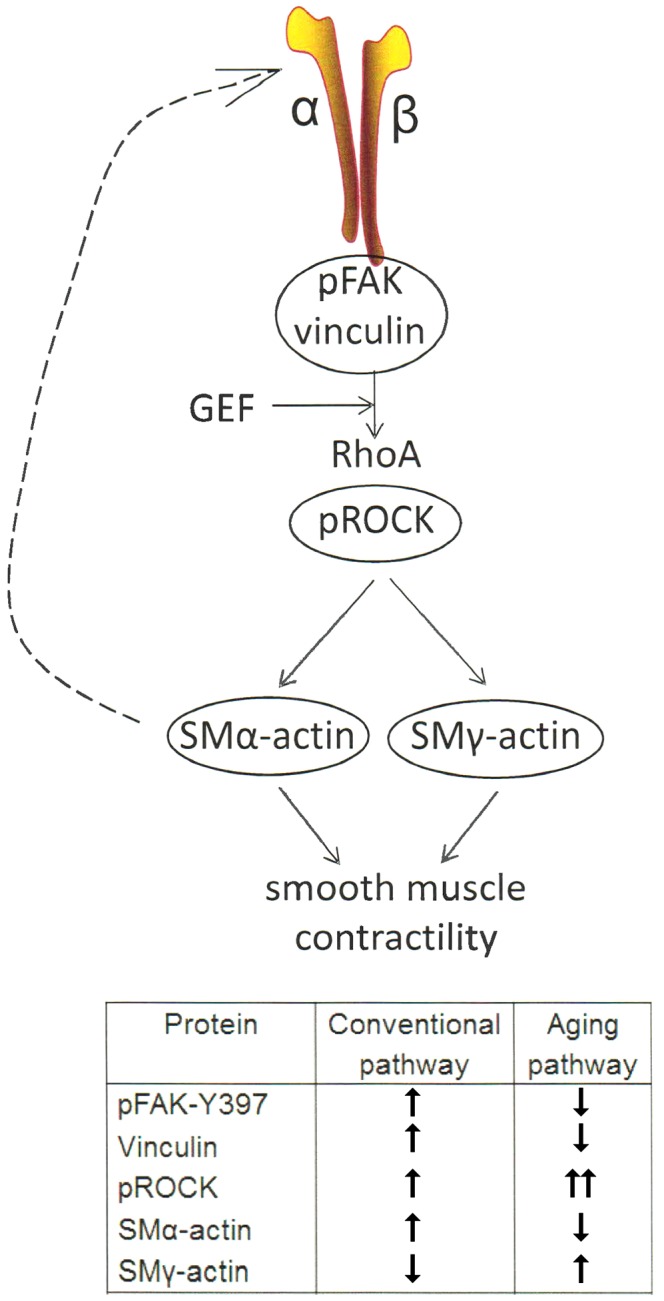
One of the main mechanotransduction pathways in VSM cell contractility is the RhoA/ROCK regulated integrin-actomyosin axis. Mechanical signals (e.g., pressure, matrix stiffness) presented to VSM cells through integrins stimulate the activity of guanine nucleotide exchange factors (GEF) which in turn activate RhoA pathway, including ROCK which enhances actomyosin contractility. Consequently, actomyosin-induced contractility promotes VSM cell stiffening. This effect feeds back (dashed arrow) into changing cytoskeletal tension and integrin mechanosensing at the membrane level. Integrin α_5_β_1_ is known to be involved in regulating cellular and vessel contractility (Martinez-Lemus et al., 2005a). In young cells, actomyosin apparatus is strong and the VSM cell is able to properly regulate its stiffness and adhesion to the matrix in response to mechanical signals. In aging, however, the decrease in SMα-actin stress fiber formation and alteration of cell-matrix adhesions induces a reduced VSM cell contractility and deficient mechanosensing. We suggest that this dysregulation induces compensatory effects by up-regulating ROCK and SMγ-actin which increases cell stiffness without regaining contractile properties of the cell (see table inset).

In summary, the results of this study demonstrate that age-induced changes at the molecular level in VSM cells play a central role in reduced contractile function of SFA (**Figure 6**). Our data support a synthetic phenotype for old cells with reduced mechanosensitivity and adaptation to mechanical signals from their microenvironment.

## Author Contributions

JS, HG, AY, CC, CW, and AT designed the experiments. JS, HS, HG, SP, and SS performed the experiments. JS, HS, HG, and JT analyzed the data. JS, HS, HG, JT, CW, and AT wrote the manuscript.

## Conflict of Interest Statement

The authors declare that the research was conducted in the absence of any commercial or financial relationships that could be construed as a potential conflict of interest. The reviewer EB and handling Editor declared their shared affiliation.
